# School-based strategies for oral health education of adolescents- a cluster randomized controlled trial

**DOI:** 10.1186/1472-6831-12-54

**Published:** 2012-12-18

**Authors:** Abdul Haleem, Muhammad Irfanullah Siddiqui, Ayyaz Ali Khan

**Affiliations:** 1 Department of Oral Health Sciences, Federal Postgraduate Medical Institute, Shaikh Zayed Medical Complex, Lahore, 54600, Pakistan; 2 Department of Community Medicine and Pilgrims, Umm Al-Qura University, Makkah Al-Mukarrama, 7607, Saudi Arabia

**Keywords:** Oral health education, Dental health education, Oral health promotion, Prevention, Oral diseases, Peer-led, Teacher-led, Dentist-led

## Abstract

**Background:**

Oral health education (OHE) in schools has largely been imparted by dental professionals. Considering the substantial cost of this expert-led approach, the strategies relying on teachers, peer-leaders and learners themselves have also been utilized. However the evidence for comparative effectiveness of these strategies is lacking in the dental literature. The present study was conducted to compare the effectiveness of dentist-led, teacher-led, peer-led and self-learning strategies of oral health education.

**Methods:**

A two-year cluster randomized controlled trial following a parallel design was conducted. It involved five groups of adolescents aged 10-11 years at the start of the study. The trial involved process as well as four outcome evaluations. The present paper discusses the findings of the study pertaining to the baseline and final outcome evaluation, both comprising of a self-administered questionnaire, a structured interview and clinical oral examination. The data were analyzed using Generalized Estimating Equations.

**Results:**

All the three educator-led strategies of OHE had statistically higher mean oral health knowledge (OHK), oral health behavior (OHB), oral hygiene status (OHS) and combined knowledge, behavior and oral hygiene status (KBS) scores than the self-learning and control groups (p<0.001). The mean OHK, OHS and KBS scores of the three educator-led strategies did not differ significantly. The peer-led strategy was, however, found to have a significantly better OHB score than the respective score of the teacher-led strategy (p<0.05). The self-learning group had significantly higher OHB score than the control group (p<0.05) but the OHK, OHS and KBS scores of the two groups were not significantly different.

**Conclusions:**

The dentist-led, teacher-led and peer-led strategies of oral health education are equally effective in improving the oral health knowledge and oral hygiene status of adolescents. The peer-led strategy, however, is almost as effective as the dentist-led strategy and comparatively more effective than the teacher-led and self-learning strategies in improving their oral health behavior.

**Trail registration:**

SRCTN39391017

## Background

In South East Asian countries a significant proportion of adolescents are having poor oral hygiene and betel-nut chewing habit both of which have serious public health consequences [1-5]. The former may predispose adolescents to gingivitis and periodontitis while the latter may render them at risk to oral submucous fibrosis and oral cancer. Both of these problems are associated with social and behavioral factors which can be targeted by appropriately designed school-based oral health education that may enable adolescents to make informed health-related choices as well as precipitate a health-enhancing social environment. It may also set the agenda for structural, social and political changes required to eradicate the root causes of oral health related problems in developing countries.

Traditionally oral health education (OHE) in schools has largely been imparted by dentists or dental hygienists. However the cost-effectiveness and sustainability of such an approach is questionable [6]. There are instances where the utilization of teachers for delivering and reinforcing OHE messages has been found to be feasible and effective [7,8]. But the shortage of time and heavy workload at schools have been cited as important factors that adversely affect the effectiveness of teachers as oral health educators [9].

Another school-based resource person whose potential has been exploited in OHE programs is a trained peer of school children. There is a growing body of research that shows that the school-based peer-led health education is more effective than the teacher-led [10,11] and at least as effective as the expert-led [10] health education. However the evidence for the comparative effectiveness of the three strategies is lacking in the dental literature. The present study was conducted to compare the effectiveness of OHE strategies relying on dentist, teacher, peer leader and the learner himself/ herself as resource persons.

### Null hypothesis

The dentist-led, teacher-led, peer-led and self-learning strategies of oral health education are not significantly different from one another as well as from the control group in increasing knowledge about oral health; and in bringing about a positive change in oral health behavior and oral hygiene status of school children aged 10-11 years.

## Methods

### Trial design and study participants

**Figure 1:**
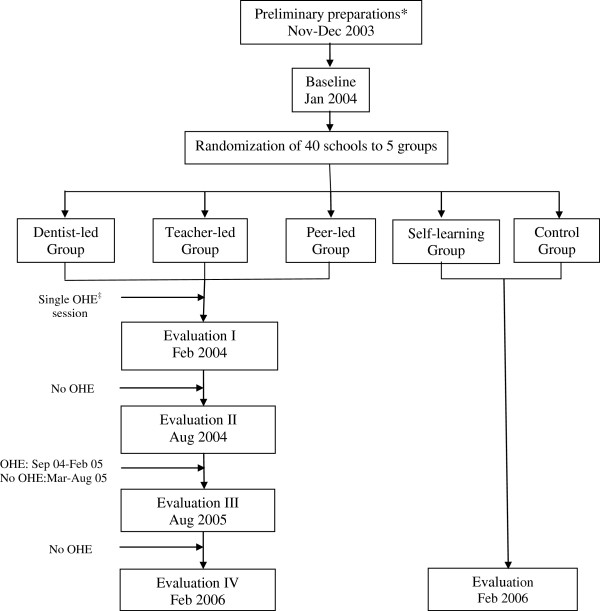
Schematic diagram of OHE component of the Preventive Oral Health Care Project.^*^Permissions; and designing and testing of OHE material, questionnaire, clinical forms; training of educators. ^‡^Oral Health Education.

The trial was a parallel cluster randomized controlled trial of two years duration. It made a part of a preventive oral health care project designed for adolescents, aged 10-11 years at the start of the project. The trial involved five groups of boys and girls studying in class six of forty public and private schools. Three of the study groups were imparted oral health education (OHE) by dentist, teachers or peer group leaders. The fourth group was a self-learning group whiles the fifth one that did not receive any form of OHE, served as a control group. The dentist-led (DL), teacher-led (TL) and peer-led (PL) groups were given a single educational input after the baseline data collection by the end of January 2004. Afterwards the groups did not receive any form of oral health education till August 2004. The oral health education messages were then repeated and reinforced on a monthly basis from Sep 2004 to Feb 2005. This was followed by a period of one year of no oral health education. The three educator-led groups were subjected to four evaluations during the course of the trial. Evaluation I was conducted immediately after the first education session to observe the effect of the single OHE input on the dependent variables. Evaluation II was performed approximately six months after evaluation I to measure the sustainability of the effect resulting from one-time OHE. Evaluation III and evaluation IV were undertaken six months and a year after the reinforcement phase of the project to determine the long term impact of repeated and reinforced OHE on the outcome variables of the study. The self learning and control groups were surveyed at baseline and at the end of two years. The schematic diagram of the oral health component of the preventive oral health care project is shown in Figure 1.

### Outcome measures

The primary outcome measures included improvement in oral hygiene status measured through a decrease in the number of sextants of oral cavity having dental plaque without bleeding on probing, plaque with bleeding on probing and calculus. The secondary outcome measures were oral health related knowledge and preventive oral health behavior about gingivitis and OSMF/ oral cancer.

### Ethical approval

The ethical approval for the trail was given by the Institutional Review Board of Shaikh Zayed Medical Complex, Lahore (Ref. No. SZH/IRB/017-03). The trail was registered with the Current Controlled Trails (http://www.controlled-trails.com) under the ISRCTTN number 39391017.

### Sample size

At the contemplation stage of the trial, it was presumed that the OHE interventions under investigation would result in 50% reduction in the existing prevalence of gingivitis (34%) in 12 years old urban school children in Pakistan [1]. Given an approximately equal cluster size and 80% power of the study at an α level of 0.05, the number of subjects in each study group, if they were to participate in a clinical trial using individual randomization (RCT) [12], was determined as 99. It was then adjusted for the cluster randomized controlled trial (CRT) in question by assuming an intra-class correlation coefficient (ICC) of 0.05, a design effect of 3 and about 11% loss of subjects expected over the period of the trial. The required number of study subjects in each group was thus estimated to be about 327. The number of clusters in each study group was calculated as eight with 35-45 students in each cluster [13].

### Sample selection and randomization

**Figure 2:**
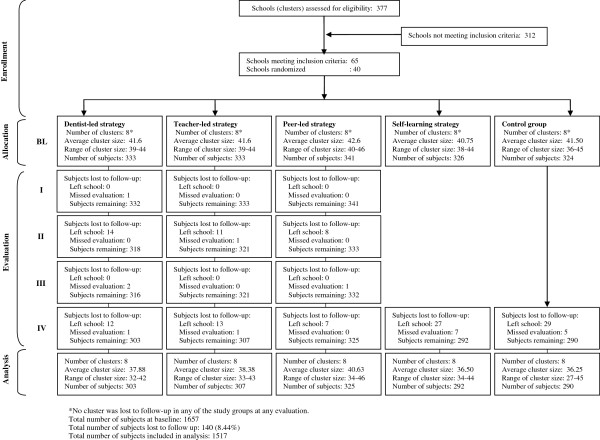
Flow chart of clusters and study subjects through different phases of the trial.

All public and private boys’ and girls’ schools having more than one section of class six and not less than thirty five students per section in the two adjacent towns of the cosmopolitan city of Karachi, Pakistan were eligible to participate. The said towns were chosen because of their socio-economic and ethnic homogeneity. A total of 377 schools were assessed for eligibility, 124 public and 253 private (Figure 2). Public schools had distinct categories of boys' (n = 75) and girls' (n = 49) schools but all private schools had co-education. The latter, however, had separate sections for boys and girls in classes from six to ten. Three hundred and twelve schools did not meet the eligibility criteria leaving behind 65 schools. Twenty public schools, ten each from the girls' and boys' categories of public schools, were randomly chosen for the study followed by a random selection of one section of class six in each of these schools to participate in the study. From amongst the private schools fulfilling the eligibility criteria, a total of twenty schools were chosen at random but in ten of these schools a boys’ section of class six each while in the other ten a girls’ section was randomly selected. Schools were considered as the units of randomization to cause minimum disruption of the school routine and to prevent the contamination of OHE strategies. The parents of all children to be involved in the project were sent introductory letters accompanied by the consent forms through the school principals. All parents gave a positive consent.

All selected schools in the public-private strata and boys’-girls’ sub-strata were assigned serial numbers by a teacher not involved in the project. The teacher then used a lottery method to randomly allocate two boys’ and two girls’ schools each from the respective lists of public and private schools to each of the five study groups.

### Selection and training of educators

In schools selected for teacher-led (TL) strategy, the teachers-in-charge of the selected sections of class six were assigned the responsibility of OHE. In case of peer-led (PL) strategy one student in each selected section was nominated as peer leader by the teacher-in-charge depending on his/ her academic record, regularity in attendance and the ability to socialize. For dentist-led (DL) strategy, a community dentist was chosen.

The selected dentist, teachers and peer leaders were trained for OHE by organizing five 2-hour sessions. The training was highly structured and based on a written set of learning objectives and activities for each session. Pre- and post-training assessment of the oral health knowledge and attitudes of the educators towards OHE of students was undertaken. The training included a live demonstration of an OHE session taken by the first author. After training all educators practiced taking an OHE session each in their respective schools in a section of class six not included in the main study. Every educator was then evaluated by the first author using a ten-point check list while taking another OHE session. The points included in the check list were: a brief description of the importance of oral health and the objectives of the OHE session, a full coverage of the OHE contents in a sequential manner, proper use of posters, the use of layman language, ensuring active participation and appreciation of a positive behavior, timely completion of the session allowing time for questioning.

### Oral health education interventions

OHE comprising of a one-hour session in all strategies was based on the contents of a booklet supplemented by a set of seven pre-tested posters and an instruction manual for the educators (all three designed and pilot tested for the project). The session included twenty minutes of oral health education, five minutes of brushing demonstration, five minutes of question-answer and thirty minutes of group activities.

OHE during each session covered six main topics. The first introductory topic reminded adolescents of the functional and psychosocial role of healthy teeth and the importance of taking care of them. The second topic about human dentition dealt with some practical aspects of the anatomy of a tooth (e.g. enamel is insensitive while dentine and cementum are sensitive), the number of deciduous and permanent teeth and the approximate age of eruption of permanent molars. The third and fourth topics discussed the natural history of dental caries, chronic gingivitis and periodontitis including their important signs and symptoms. The fifth topic informed students about various betel nut and tobacco containing products available in the society as well as the health consequences of using these products. The last topic covered the caries preventive measures including reduced amount and frequency of sugary foods, consuming protective foods e.g. cheese, peanuts as snacks and twice daily use of fluoride tooth paste. The purpose (removing plaque thoroughly from all tooth surfaces and cervical areas of teeth to prevent gingivitis) and the systematic method of tooth cleaning was explained and demonstrated using a tooth brush and a model of teeth. The OHE session ended up with a brief discussion about how to have a betel nut/ tobacco free environment in schools and in the society.

The theoretical framework of OHE in the three educator-led strategies was based on the constructs of social cognitive theory including vicarious learning, active participation, skill training, self efficacy, reinforcement and social support [14]. These constructs were made to operate by getting students involved in various group activities including examining one another’s teeth for dental plaque and calculus by tooth picks and looking for cavities (vicarious learning and active participation), practicing and demonstrating tooth brushing to one another on models of teeth and identifying cariogenic and carcinogenic eatables (skill training and self efficacy). The students were encouraged to appreciate their peers for taking an active part in the above mentioned activities and for a desirable change in their oral health behavior (reinforcement and social support). They were given OHE booklets to take home and asked to share their knowledge and skills with their family members.

In DL and TL strategies the educators took an OHE session with all children sitting in their respective sections. After the lecture and tooth cleaning demonstration, they divided the students into five groups of seven to nine students each and asked them to carry out group activities. In the meanwhile they kept moving from one group to another to maintain discipline in the class.

In PL strategy students in each section were divided into five groups and an OHE session was conducted with each group in a separate room. The peer leader initiated and facilitated discussion about oral diseases and their prevention, and supervised the students’ activities. Two such sessions per day were arranged in each section till all children were covered in that section.

Considering the involvement of non-dental personnel, the OHE intervention was designed to be simple, objective and easy to deliver. The objectives of oral health education were clearly defined and its essential elements stated explicitly in the instruction manual for the educators. The latter also contained a standardized format to be followed by all educators as well as a written description of different illustrations in every poster in a sequential order. The educators were trained and encouraged to follow this sequence throughout the program to ensure full coverage of the contents of the OHE booklet in all sessions.

The children in the self-learning (SL) group were given the OHE booklet to read while the control (CL) group did not receive any form of OHE.

### Process evaluation

The first author observed three sessions each of the teachers, the peer leaders and the dentist during implementation of the OHE program and scored them for fidelity (range: 0-10) using the ten-point check list. The first observation was undertaken during the one-time OHE session while the second and third observations were made in the beginning and the middle of the reinforcement phase of the program. The fidelity scores of different educators ranged from eight to ten. Every observed session was followed by a feedback provided to the educators.

In addition two focus group discussions, one following one-time OHE and the second at the end of the repetition and reinforcement phase of the project formed a part of the feedback and process evaluation mechanisms.

### Data collection methods

The data related to the outcome measures, at baseline and the following evaluations, were collected through a self-administered questionnaire, a structured interview and a clinical oral examination of the study participants.

The questionnaire included twelve close-ended questions about oral health knowledge and four about oral health behavior. The oral health knowledge questions included questions about the number of deciduous and permanent teeth; the age of eruption of first molar tooth, the most important signs; causes and prevention of dental caries and gingivitis; and the health hazards of betel nuts and tobacco. The questions related to oral health behavior inquired about the method and frequency of tooth cleaning as well as the use of betel nuts and tobacco products. Most of the questions were based on the established scientific facts in OHE such as caries preventive effect of fluoride, etiological role of dental plaque in gingivitis, role of twice daily frequency and thoroughness of tooth cleaning in preventing gingivitis, cariogenecity of sugar and refined starches, and carcinogenic potential of tobacco and betel nuts [15-17]. Some questions evolved from a previous KAP (knowledge, attitude and practice) study on class six students and focus group discussions with teachers and dentists in Karachi (unpublished data). These investigations pointed out a lack of knowledge of the study participants about the number of milk teeth. Moreover all students and teachers considered first permanent molar a milk tooth since it erupted without exfoliation of a deciduous predecessor, and preferred to leave it untreated or to have it extracted if affected by caries.

The questionnaire was filled by the study participants in each section in the presence of teacher-in-charge who replicated the examination conditions to prevent interaction of students during filling out of the questionnaire.

The structured interview, conducted by a trained dental assistant before questionnaire survey, comprised of eight oral health behavior questions, five of which were about the practices of adolescents with regard to buying and sharing of commonly used eatables (the respondents were asked to pick their favorites jumbled up on a table), thoroughness of cleaning teeth and their cervical areas (adolescents were required to demonstrate on the model of teeth); and making sure that one uses fluoride while cleaning teeth (the participants were expected to look for the contents of the tooth paste or pick fluoride-containing miswak, both lying on the table). Two questions explored the stated behavior of adolescents towards keeping the company of peers with bad breath and of those using betel-nut containing products. The last question was about the adolescents’ stated role in persuading their peers to avoid/ quit the use of betel-nuts. All questions for the interview were having ‘right-wrong’ and ‘yes-no’ type of responses.

The face and content validity of the questions was examined by a panel of six researchers, two each from the fields of community medicine and dentistry and two educationists, based on defined and mutually agreed criteria. The reliability of questions was assessed by a test-retest study on a group of forty class six adolescents and calculating the Pearson’s reliability coefficient (r) which was found to be 0.79 for the questionnaire and 0.87 for the interview questions. The triangulation technique was used to validate questions about self reported behavior by cross checking the responses with the findings of oral examination and by observing the behavior during the interview phase of the test-retest study. The values of ‘r’ as a measure of criterion validity of reported frequency of tooth brushing against the clinical measure of gingival bleeding, demonstrated behaviors of thorough tooth cleaning and cleaning of cervical areas of teeth were 0.67, 0.81, and 0.84 respectively. The reported behaviour of consuming betel nut products was found to be highly correlated to the common practices of adolescents of buying (r=0.89) and sharing (r=0.91) these products with their peers disclosed during the structured interview.

The clinical oral examination of all study subjects was performed at baseline and subsequent evaluations by a trained and calibrated dentist. Teeth were examined for plaque, gingival bleeding on probing and calculus. The examination was carried out by employing a plain mouth mirror and Community Periodontal Index (CPI) probe under natural light coming through a window. During examination subjects were seated in an ordinary plastic chair facing the window while the examiner seated on the right side of the subjects on an examination stool with adjustable height. The mouth mirror was used for indirect visualization of teeth in the upper dental arch, to enhance illumination of teeth by reflecting light on them and to retract cheeks and lips. The CPI probe was used to confirm the presence of plaque and calculus visible to the naked eye by running the probe along the gingival margin from mesial to distal surface on labial/ buccal as well as on the lingual/ palatal side of the index teeth (the two central incisors and the two most posterior teeth on the right and left sides of the upper and lower dental arches). The bleeding on gentle probing was also detected by inserting the probe into the gingival sulcus with a force of not exceeding 20 grams (the amount of force that does not cause blanching when probe tip is inserted under the nail bed) and withdrawing it parallel to and keeping its tip in contact with the tooth surface. The procedure was repeated on three sites (mesial, mid-labial/ buccal , distal) on both the labial/ buccal and lingual/ palatal surfaces of the index teeth. The resistance felt while withdrawing the probe showed the presence of calculus lying in the gingival sulcus/ along the gingival margin; and bleeding indicated gingivitis. A sterilized set of mirror and probe was used for every child and all instruments were cleaned, disinfected and autoclaved before they were reused.

A standard format for clinical examination was used. The oral cavity was divided into six sextants: right upper posterior (from first premolar to second molar tooth), upper anterior (from right canine to left canine tooth), left upper posterior, left lower posterior, lower anterior and right lower posterior. The sextants were examined in a clock-wise direction taking a start from right upper posterior sextant and ending at the right lower one. The buccal/ labial surfaces of teeth were examined first followed by examination of lingual/ palatal surfaces. A dichotomous scale with ‘present’ and ‘absent’ categories was used for plaque, gingival bleeding and calculus. Dental plaque and calculus were scored as present if visible to the naked eye as well as detected by CPI probe. A note of bleeding was taken provided it was associated with accumulation of visible plaque along the gingival margin. The findings were recorded by a recorder (seated on the left side of the examining subjects close enough to listen to the examiner clearly) on a specially designed form. The oral hygiene codes which were entered in the recording form included: 0 = no plaque, 1 = plaque without gingival bleeding, 2 = plaque with gingival bleeding and 3 = calculus. The worst oral hygiene code for any of the two index teeth in each sextant was recorded.

5% of total children in randomly selected schools were re-examined at baseline and all evaluations on the same day to have a check on the examiner’s reliability assessed by applying Kappa test. The Kappa values for these re-examinations were always well above 0.8 [18,19].

### Blinding

The dentist and the dental assistant who conducted oral examination and the structured interview respectively were kept blind to the group allocation of the study subjects right from baseline till the end of the study. Similarly, the names of the schools and their allocation to different OHE strategies were concealed by assigning numbers (1-40) to schools and alphabets (A-E) to the strategies at random to ensure blinding of the data entry operator and data analyst. Although, the educating dentist, teachers and peers-leaders were trained together, the participants of the training program as well as the head-teachers and administrators of the participating schools were requested in an introductory session of the project not to disclose to their students that a similar program is going on in other schools of the area as well. Furthermore, the students were kept unaware of the schedule of different activities of the project.

### Data organization and analyses

The responses to all questions were dichotomized as either correct or incorrect. The correct responses were given a score of one each and incorrect zero. The questions were categorized in two domains, OHK (oral health knowledge) and OHB (oral health behavior). The mean score for each domain was then estimated. In addition the scores for five questions related to knowledge about gingivitis and oral cancer were combined to form KGO (knowledge about gingivitis and oral cancer/ oral submucous fibrosis) index.

The clinical data were organized to estimate the number of sextants of oral cavity having dental plaque without bleeding on probing (PLQ), plaque with bleeding on probing (BOP) and calculus (CAL). Subsequently the individual scores of these three indicators were first added and then subtracted from twelve (the total number of sextants of oral cavity: six lingual/ buccal and six labial/ palatal) to produce an oral hygiene index (OHS). The OHS reflected the number of sextants of dentition free of dental plaque, bleeding on probing and calculus.

Finally an additive index KBS was constructed by combining OHK, OHB and OHS scores. The OHK, OHB and OHS scores had a range of 0 to12 each while the scores of the KGO and KBS indices ranged from 0 to 5 and 0 to 36 respectively.

The data were analyzed using the SPSS 16 program. The mean OHK, KGO, OHB, OHS, KBS, PLQ, BOP and CAL scores of the study groups were compared using Generalized Estimating Equations (GEE) [20] with log link function and exchangeable correlation matrix to account for the clustering effect. ‘Strategy’, ‘sex’ and ‘type of school’ were used as independent variables in the model. The minimum level of statistical significance for comparing the study groups was set at p<0.05. However this chosen α-level was adjusted for multiple comparisons by applying Bonferroni correction in GEE model. The risk ratios (effect sizes) obtained from GEE analysis were exponentiated to make them more meaningful. The percent change from baseline to evaluation IV in the adjusted mean scores of different indices was also calculated as this has been one of the commonly used measures in studies previously reported in the dental literature [7,21-23].

## Results

The study started with 1657 adolescents studying in 40 randomly chosen schools in the study area. The data of 140 children were excluded from the final analysis as 121 children left their respective schools before the completion of the study while 19 children missed one or more evaluations. Therefore data of 1517 children who completed all evaluations conducted in their respective groups were subjected to analyses. Table 1 shows the distribution of the study subjects according to sex and type of school. It also presents the number and percentage of the study subjects who were lost to follow up over the period of the study. It can be observed that the total loss was about 8.5%, the lowest for the peer-led strategy (4.7%) and the highest for the control group (10.5%). The baseline data of the study subjects who dropped out and of those who continued were subjected to Chi-square and independent sample t-tests. No statistically significant differences were found between the two groups of subjects at baseline with regard to gender, type of school, oral health knowledge, attitudes, behaviour, plaque without bleeding on probing, plaque with bleeding on probing and calculus.

**Table 1:** Distribution of the study subjects (No. & %) according to gender and type of school (n=1517)

		**OHE Strategy**					
**School**	**Sex**	**DL**	**TL**	**PL**	**SL**	**CL**	**Total**
Public	Male	81 (52.6)	69 (44.8)	81 (47.6)	81 (54.4)	81 (50.6)	393 (49.9)
	Female	73 (47.4)	85 (55.2)	89 (42.4)	68 (45.6)	79 (49.4)	394 (50.1)
	Total	154 (50.8)	154 (50.2)	170 (52.3)	149 (51.0)	160 (55.2)	787 (51.9)
Private	Male	70 (47.0)	76 (49.7)	72 (46.5)	74 (51.7)	60 (46.2)	352 (48.2)
	Female	79 (53.0)	77 (50.3)	83 (53.5)	69 (48.3)	70 (53.8)	378 (51.8)
	Total	149 (49.2)	153 (49.8)	155 (47.7)	143 (49.0)	130 (44.8)	730 (48.1)
	Grand total	303 (20.0)	307 (20.2)	325 (21.4)	292 (19.2)	290 (19.1)	1517 (100.0)
	LTF	30 (9.0)	26 (7.8)	16 (4.7)	34 (10.4)	34 (10.5)	140 (8.5)

*OHE*: Oral health education, *DL*: Dentist-led, *TL*: Teacher-led, *PL*: Peer-led, *SL*: Self-learning strategies of OHE, *CL*: Control group.

*DL*: Dentist-led, *TL*: Teacher-led, *PL*: Peer-led, *SL*: Self-learning strategies of OHE, *CL*: Control group.

*LTF*: Lost to follow-up.

The present paper presents the results of the study pertaining to the baseline (BL) and final outcome evaluation (IV) scores of the study groups.

Table 2 shows the adjusted mean OHK, KGO, OHB, OHS, KBS, PLQ, BOP and CAL scores of the study subjects at baseline and evaluation IV. All evaluation IV scores of the three educator-led groups were statistically higher than their corresponding scores at baseline (p<0.001). The SL group had statistically higher evaluation IV OHK, OHS, PLQ scores (p<0.001) as well as OHB, KGO and CAL scores (p<0.05) than the respective scores at baseline. This group, however, had insignificantly different KBS and BOP baseline and evaluation IV scores. The evaluation IV scores of the CL group were statistically better than the baseline scores only in case of OHK, KGO and CAL indices (p<0.05).

**Table 2:** Adjusted mean scores (95% confidence interval) at baseline (BL) and final evaluation (IV)

		**ICC**^**‡**^	**Dentist-led**	**Teacher-led**	**Peer-led**	**Self-learning**	**Control**
OHK^a^	BL	0.05	2.29 (2.05-2.54)	2.59 (2.34-2.83)	2.36 (2.12-2.60)	2.40 (2.15-2.64)	2.31 (2.07-2.56)
	IV	0.25	5.33 (4.80-5.87)	5.32 (4.79-5.85)	5.58 (5.05-6.11)	3.14 (2.60-3.67)	2.80 (2.26-3.34)
KGO^b^	BL	0.01	0.63 (0.54-0.71)	0.67 (0.59-0.75)	0.63 (0.55-0.72)	0.65 (0.56-0.74)	0.58 (0.49-0.66)
	IV	0.22	2.21 (1.91-2.52)	1.70 (1.39-2.00)	2.22 (1.91-2.52)	0.86 (0.55-1.17)	0.82 (0.51-1.13)
OHB^c^	BL	0.04	3.39 (3.30-3.69)	3.59 (3.29-3.89)	3.42 (3.12-3.71)	3.54 (3.24-3.85)	3.26 (2.95-3.57)
	IV	0.42	7.30 (6.97-7.47)	7.14 (6.82-7.47)	7.92 (7.60-8.24)	4.14 (3.81-4.47)	3.34 (3.01-3.69)
OHS^d^	BL	0.09	4.20 (3.52-4.88)	3.49 (2.81-4.16)	3.46 (2.79-4.13)	5.22 (4.53-5.91)	3.52 (2.82-4.21)
	IV	0.10	5.20 (4.66-5.74)	4.86 (4.32-5.39)	5.00 (4.47-5.53)	4.02 (3.47-4.56)	3.30 (2.76-3.84)
PLQ^e^	BL	0.11	5.02 (4.55-5.48)	5.48 (5.02-5.94)	5.23 (4.78-5.68)	3.44 (2.98-3.90)	4.91 (4.44-5.38)
	IV	0.07	3.77 (3.29-4.25)	4.33 (3.85-4.81)	4.22 (3.74-4.69)	4.21 (3.73-4.70)	4.64 (4.16-5.13)
BOP^f^	BL	0.06	2.25 (1.82-2.68)	2.23 (1.80-2.65)	2.34 (1.91-2.76)	2.18 (1.74-2.61)	2.30 (1.86-2.73)
	IV	0.11	1.59 (1.27-1.92)	1.18 (0.86-1.50)	1.06 (0.75-1.38)	2.19 (1.87-2.52)	2.52 (2.19-2.85)
CAL^g^	BL	0.03	0.70 (0.52-0.88)	0.73 (0.55-0.91)	0.91 (0.73-1.08)	0.95 (0.77-1.13)	1.16 (0.98-1.34)
	IV	0.03	1.51 (1.25-1.76)	1.63 (1.38-1.88)	1.52 (1.27-1.76)	1.34 (1.08-1.60)	1.46 (1.21-1.72)
KBS^h^	BL	0.09	9.85 (8.86-10.85)	9.68 (8.69-10.67)	9.23 (8.25-10.21)	11.20 (10.20-12.20)	9.13 (8.13-10.14)
	IV	0.34	17.80 (16.70-18.90)	17.35 (16.26-18.45)	18.56 (17.48-19.65)	11.34 (10.23-12.44)	9.54 (8.43-10.64)

Note: All scores adjusted for sex, type of school and clustering effect using Generalized Estimating Equations, ‡Intraclass correlation coefficient.

^a^Oral health knowledge (total score: 12); ^b^Knowledge about gingivitis and oral cancer (total score: 5); ^c^Oral health behavior (total score: 12).

^d^Oral hygiene status (total score: 12, indicates the number of sextants of oral cavity free of dental plaque, bleeding on probing and calculus).

^e^Plaque (total score: 12); ^f^Bleeding on probing (total score: 12); ^g^Calculus (total score: 12); ^h^Oral health knowledge, oral health behavior and oral hygiene status (total score: 36).

The percent change in the mean scores of the study groups along with the significant group differences at baseline and final evaluation are depicted in Table 3. The study results showed an increase of about 23-27% in the OHK score of adolescents in the three educator-led groups, around 21-32% gain in their KGO score, an approximate increase of 30-37% in their OHB score, an improvement of about 8-13% in their OHS score, a 21-26% increase in their KBS score, a decrease of about 8-10% in their PLQ score and an approximate reduction of 5-11% in BOP score of these groups. The CAL scores of all study groups increased from baseline to final evaluation by 2-8%. The SL group showed an approximate increase of 4-6% in OHK, KGO and OHB scores with a negligible change in its BOP and KBS scores. The PLQ score of this group showed an increase of about 6% while its OHS score deteriorated by 10% from baseline to final evaluation. The OHK and KGO scores of the CL group showed an increase of about 4-5% from baseline to final evaluation while its OHB and KBS scores improved by only about 1%. The PLQ and OHS scores of this group decreased but its BOP score increased by about 2% from baseline to evaluation IV.

**Table 3:** **Percent change in adjusted mean scores**^**†**^**and significant group differences at baseline (BL) & evaluation IV**

	**Percent change**					**Significant group differences**
	**DL**	**TL**	**PL**	**SL**	**CL**	**p-values**
OHK^a^	25.33	22.75	26.83	6.17	4.08	IV: DL, TL, PL > SL, CL^**^
KGO^b^	31.60	20.40	31.80	4.20	4.80	IV: DL, TL, PL > SL, CL^**^
OHB^c^	32.58	29.58	37.50	5.00	0.67	IV: DL, TL, PL > SL, CL^**^; PL > TL^*^; SL > CL^*^
OHS^d^	8.33	11.42	12.59	−10.00	−1.83	BL: SL > DL, TL, PL, CL^*^; IV: DL, TL ,PL > SL, CL^**^
PLQ^e‡^	−10.42	−9.58	−8.42	6.42	−2.25	BL: SL > DL, TL, PL, CL^*^; IV: DL, TL, PL > SL, CL^**^
BOP^f‡^	−5.50	−8.75	−10.67	0.08	1.83	IV: DL, TL, PL > CL^**^, TL, PL > SL^**^, DL>SL^*^
CAL^g‡^	6.75	7.50	5.09	3.25	2.50	BL: DL, TL > CL^*^
KBS^h^	22.08	21.30	25.92	0.39	1.14	BL: SL > CL (p=.042), IV: DL, TL, PL > SL, CL^**^

^†^All scores adjusted for sex, type of school and clustering effect using Generalized Estimating Equations.

DL: Dentist-led; TL: Teacher-led; PL: Peer-led; SL: Self-learning; CL: Control groups.

^a^Oral health knowledge (total score: 12); ^b^Knowledge about gingivitis and oral cancer (total score: 5); ^c^Oral health behavior (total score: 12).

^d^Oral hygiene status (total score: 12, indicates the number of sextants of oral cavity free of plaque, bleeding on probing and calculus).

^e^Plaque (total score: 12); ^f^Bleeding on probing (total score: 12); ^g^Calculus (total score: 12) (^‡^A negative sign associated with percent change shows a decrease and an improvement in score and vice versa for a positive sign, and a statistically better score has a lower value).

^h^Oral health knowledge, oral health behavior and oral hygiene status (total score: 36); ^*^P<.05; ^**^P<.001.

The study groups did not have statistically significant differences at baseline with regard to OHK, KGO, OHB, KBS and BOP scores. The SL group, however, had a marginally better baseline KBS score than the CL group (p=0.042) (Table 3). This group also had a significantly lower baseline PLQ score (p< 0.001) and consequently had a statistically better baseline OHS score than the other four groups (p<0.05). The CAL baseline score of the CL group was significantly higher than the corresponding scores of the DL and the TL groups (p<0.05).

The educator-led groups had insignificantly different OHK, KGO, OHB, OHS, KBS, PLQ, BOP and CAL scores at evaluation IV with only one exception in which case the PL group had a statistically higher OHB score than the TL group (p<0.05). All these scores of the educator-led groups were significantly better than the respective scores of SL and CL groups at p<0.001 except the BOP score of the DL group which was significantly better than that of the SL group at p<0.05. The SL and CL groups exhibited a statistically significant difference in favor of the SL group only in case of the OHB index score at evaluation IV (p<0.05).

The effect sizes of different education strategies with reference to the control group (effect size=1) are presented in Table 4. The effect sizes having the lower values in case of PLQ, BOP and CAL indices were considered superior to those with the higher values. It can be seen in this table that the educator-led strategies had the greatest impact on the KGO index and the smallest effect on the CAL index. The effect sizes of the PL strategy in case of most of the indices were comparatively greater than those of the other education groups. This strategy had a substantial effect on the knowledge (KGO) and preventive behavior (OHB) of adolescents about gingivitis and oral cancer.

**Table 4:** Effect sizes (β)* of different OHE strategies (95% CI) at evaluation IV

	**Dentist-led**	**Teacher-led**	**Peer-led**	**Self-learning**
OHK^a^	1.90 (1.53-2.36)	1.90 (1.53-2.36)	1.99 (1.61-2.47)	1.12 (0.87-1.45)
KGO^b^	2.77 (1.86-4.13)	2.12 (1.40-3.20)	2.79 (1.87-4.15)	1.08 (0.65-1.80)
OHB^c^	2.18 (1.96-2.43)	2.14 (1.92-2.38)	2.37 (2.13-2.64)	1.24 (1.09-1.41)
OHS^d^	1.58 (1.30-1.91)	1.47 (1.21-1.79)	1.51 (1.25-1.84)	1.22 (0.98-1.50)
PLQ^e‡^	0.81 (0.69-0.96)	0.93 (0.80-1.09)	0.91 (0.78-1.06)	0.91 (0.78-1.06)
BOP^f‡^	0.63 (0.50-0.80)	0.47 (0.35-0.63)	0.42 (0.30-0.58)	0.87 (0.71-1.06)
CAL^g‡^	1.03 (0.81-1.31)	1.12 (0.89-1.41)	1.04 (0.82-1.32)	0.92 (0.71-1.19)
KBS^h^	1.87 (1.64-2.13)	1.82 (1.59-2.08)	1.95 (1.71-2.22)	1.19 (1.02-1.38)

*The estimates are the exponentiated risk ratios (RR for control group: 1). All values adjusted for sex, type of school and clustering effect using Generalized Estimating Equations.

*OHE*: Oral health education; *CI*: Confidence Interval.

^a^Oral health knowledge (total score: 12).

^b^Knowledge about gingivitis and oral cancer (total score: 5).

^c^Oral health behavior (total score: 12).

^d^Oral hygiene status (total score: 12, indicates the number of sextants of oral cavity free of plaque, bleeding on probing and calculus).

^e^Plaque (total score: 12); ^f^Bleeding on probing (total score: 12).

^g^Calculus (total score: 12).

^h^Oral health knowledge, oral health behavior and oral hygiene status (total score: 36).

^‡^The lower the value the better it is.

## Discussion

The present paper discusses the results of the trial obtained at baseline and final evaluation to compare the study groups with regard to the outcome variables of the trial. Although the educator-led groups were subjected to three more evaluations in between, the findings of these evaluations will be utilized to discuss the role of repetition and reinforcement in oral health education in a subsequent publication of the authors.

The results of the present study showed that the three educator-led strategies were not significantly different in improving the oral health knowledge and oral hygiene status of the study participants. The adolescents in the peer-led group, however, exhibited statistically better oral health behavior than their counterparts in the teacher-led group. All three educator-led strategies of oral health education investigated in the study proved to be more effective in enhancing oral health knowledge, behavior, and oral hygiene status of the study subjects when compared with the strategies based on the options of ‘self-learning’ or ‘imparting no education at all’.

A MEDLINE search was conducted through PubMed to find out school-based studies that compared two or more OHE strategies tested in the present study. Different combinations of the keywords (along with Boolean operators) used for the search included ‘oral’, ‘dental’, ‘health education’, ‘health promotion’, ‘dentist’, ‘teacher’, ‘peer’, ’dentist-led’, ‘teacher-led’, ‘peer-led’, self-learning’, ‘school’, ‘school-based’. The website ‘http://www.pakmedinet.com’ was explored to search for the relevant articles published in the local journals. The reference lists of previous reviews of dental/ oral health education/ promotion programs were also screened for any pertinent papers [6,22-29]. The medical literature was also searched for any reviews of school-based health education interventions employing the education strategies covered by the present study.

The search of the dental literature revealed one study [30] that compared the peer-led with the dentist-led and self-teaching strategies of OHE implemented in three different secondary schools. In contradiction to the present study, all the three OHE strategies compared in that study were ineffective in increasing pupils’ knowledge about appropriate oral self care methods. However the peer education was found to be the most effective in enabling male students recognize the essential signs of gingivitis. Furthermore the pupils’ attitudes and opinions about the method of education were the most positive in the peer-led group. The dentist-led method was also very well accepted and the children in that group reported being encouraged more often to practice good oral health habits than those in the other two groups. As pointed out by the authors, the results of that study might have been influenced to a greater extent by different modes of delivery of OHE rather than the educators. The difference between the outcome measures used in that study and the present one precluded the possibility of a meaningful comparison. Nevertheless the peer-led and dentist-led methods of oral health education were shown to perform better than the self-learning strategy in both these studies.

One significant finding of the present study had been that the peer-led strategy was almost as effective as the dentist-led strategy and comparatively more effective than the teacher-led and self- learning strategies of OHE strategies in improving preventive oral health behavior of adolescents about gingivitis and oral cancer. It was also relatively better in reducing the level of gingivitis in adolescents as compared to the other education strategies. Since the issues of ‘poor oral hygiene causing gingivitis’ and ‘consumption of betel-nut containing products’ have been shown to be strongly influenced by the peer group pressures in adolescence [31-33], these findings may be the result of positive peer group norms harnessed by the peer-led strategy in the study under discussion.

The finding of a review of thirteen comparative studies of peer-led and adult-led school health education by Mellanby et al [10] that the peer-led strategy is more effective than the adult-led strategy in improving health related behavior is substantiated by the finding of the present study. Nevertheless in a meta-analysis of twelve peer-led and adult-led school-based drug prevention programs, eleven of which were included in Mellanby’s et al review [10], Cuijpers [11] concluded that it is not the leader but some other factors like contents, number of booster sessions, age of the participants and their degree of involvement that play a decisive role in determining the effectiveness of health education programs. The present study, however, tried to ensure comparability of the three educator-led groups with regard to all these factors. The present study landed support to the findings of the meta-analysis by Cuijpers [11] which showed that the peer-led drug prevention programs were more effective than the ones led by teachers (especially when booster sessions were added to the programs) but the effectiveness of the peer-led and expert-led programs was not significantly different.

The improvement in oral health knowledge resulting from dental health education interventions has been a consistent finding in the dental literature [22-29]. In the present study the three educator-led strategies produced about a six fold increase in knowledge as compared to the control group. The percent improvement in knowledge (23-27%) achieved in the three groups is greater than the increase cited in a review of oral health education (20.1%) by Brown [22]. This increase in knowledge coincides with the finding of a quasi-experimental study of a teacher-led dental health education intervention targeting 13-14 years old adolescents (n=1092) in the U.K. and reporting an average increase of about 26% in the knowledge of the study participants [7]. The latter study also reported an improvement of 1.3% in gingivitis free margins from baseline to first evaluation conducted immediately after the completion of the initial phase of the dental health education program in a small subgroup of 145 study subjects. However when this group was further subjected to dental examinations, before and three weeks after a short reinforcement program, it showed a deterioration of about 6-7% in inflammation free margins compared to the values at baseline and first evaluation [7]. On the contrary the present study found an increase of about 8-13% in the number of sextants of oral cavity free of plaque, bleeding on probing and calculus at the final evaluation in the three educator-led groups.

The percent reduction in gingival bleeding (about 5-11%) found in the three educator-led groups in the present study was slightly lower than 12.5% decrease in the gingival bleeding score mentioned in the review by Brown [22]. However the decrease in gingival bleeding in the present study might have been underestimated as it was not recorded in case of teeth with calculus (the worst condition to be recorded ignoring the presence of plaque and gingival bleeding). The same holds true for the percent reduction in plaque resulting from the educator-led strategies in the current study (8-10%) which was markedly lower than that reported in Brown’s review (17.8%) [22]. The findings of the present study like that of the Brown’s review, however, contradicted the conclusion of a review of dental health education programs by Kay and Locker [23] that school-based dental health education programs, no matter whether led by dentists, teachers or peers, had no effect on the plaque level of the study subjects. The current study showed that the oral hygiene status of the study subjects in the dentist-led, teacher-led and peer-led groups was significantly better than that of their counterparts in the self-learning and control groups at the final evaluation.

Before drawing any conclusions, certain limitations of the study are worth mentioning. The sample size estimation in the present study was based on the prevalence of gingivitis in 12 years old children reported in the last national oral health survey in Pakistan [1] that also examined the survey sample for dental caries. The prevalence and severity of caries were, however, not considered for calculating the sample size as some previous attempts to reduce caries by oral health education interventions have been disappointing [34,35]. Also no previous estimates of cognitive or behavioral measures included in the present study were available for the study population. Furthermore at the contemplation stage of the study it was presumed that the OHE interventions tested in the present study would reduce the existing prevalence of gingivitis (34%) in the study subjects by 50%. Later on the baseline data revealed a much higher prevalence of gingivitis (about 63%) and a very high prevalence of betel nut chewing habit (87.5%) in the study participants. At that stage it was estimated that the sample size calculated for the study would suffice for detecting even a 30% decrease in the prevalence of gingivitis and betel nut chewing in the study subjects, and thus it was decided to continue with the trial. But the actual reduction that was achieved in gingivitis in the three educator-led groups was 16.3%. This might have rendered the study under powered for gingivitis. The prevalence of betel nut chewing, however, decreased by about 28.5% as a result of educator-led oral health interventions.

A note of caution must also be exercised in generalizing the results of the study as the limited resources did not allow a random selection of different towns of the ethnically and socioeconomically diverse city of Karachi. This might have introduced a selection bias in the study and jeopardized its external validity. Therefore a large scale community trial is recommended to confirm the findings of the study and to ascertain the fidelity of implementation of the oral health education strategies in question under daily life conditions.

## Conclusions

Although the three educator-led strategies had a modest effect on the outcome variables included in the study, the results provide some evidence to show that the peer-led strategy may provide a feasible and almost equally effective alternative to the traditional dentist-led strategy of oral health education.

## Competing interests

The authors did not declare any competing interests.

## Authors’ contributions

The research question was conceptualized by AH. He prepared research protocol, imparted training to the persons involved, managed the research project, analyzed the data and wrote the manuscript. MIS provided guidance in data analysis and critically reviewed the methodology and manuscript. AAK supervised the research project, managed the approval and funding aspects of the project, and refined the manuscript. All authors read and approved the final manuscript.

## Pre-publication history

The pre-publication history for this paper can be accessed here:

http://www.biomedcentral.com/1472-6831/12/54/prepub
